# Artmaking in Elementary School Art Therapy: Associations with Pre-Treatment Behavioral Problems and Therapy Outcomes

**DOI:** 10.3390/children9091277

**Published:** 2022-08-25

**Authors:** Sharon Snir

**Affiliations:** 1 MA Art Therapy Program, Tel Hai College, Upper Galilee 1220800, Israel; sharonsn@telhai.ac.il; 2 The Interdisciplinary Research Center for Arts and Spirituality, Therapy, Education and Society, Tel Hai College, Upper Galilee 1220800, Israel

**Keywords:** school arts therapies, behavioral problems, experience of art making

## Abstract

Engaging in artmaking is one of the key components of art therapy. Theoretical conceptualizations posit that artmaking is not only influenced by the mental state of the artmaker, but can also modify it. The quantitative longitudinal study reported here examined these assumptions in the context of school art therapy. Seventy-seven elementary school students in art therapy in Israel completed the Art Based Intervention Questionnaire (ABI) three times during the therapy year. Their parents and homeroom teachers reported on the students’ behavioral and emotional problems on the Child Behavior Checklist (CBCL for parents, and TRF version for teachers). The results indicated an inverse correlation between the students’ externalizing and mixed problems before starting treatment and these clients’ experiences of artmaking during the first month of therapy. A regression model for predicting gain scores on the TRF internalizing problem indices was significant, whereas the significant regression predictor was the students’ experience of artmaking at T1. These findings provide initial support for an association between the experience of artmaking and mental state, and an improvement in mental state, and are discussed in relation to the context of school art therapy.

## 1. Introduction

Child art therapy provides a protected space for self-expression, self-inquiry, and dealing with distress through artmaking in a safe relationship [1]. Studies present evidence regarding the beneficial value of art for mental well-being in children [2,3]. Clinicians and art therapy theorists posit an association between the mental and emotional state of the artmaker, and the experience of artmaking in therapy, e.g., [4]. However, the nature of this relationship is complex, and its characteristics have not been sufficiently researched. The study presented below enhances recent attempts to investigate the influence of therapeutic factors on the success of art therapy [5] through the examination of elementary school children’s experiences of artmaking in therapy, and the relationship between these experiences and their condition before therapy started and with therapy outcome measures. The overarching aim was to better understand the associations between the experience of artmaking and emotional state, and between the experience of artmaking and treatment success.

### 1.1. Artmaking, Distress and Mental Well-Being

One of the key assumptions underlying art therapy is that individuals in distress are motivated to engage in artmaking, to express and deal with their difficulties [6,7]. This assumption was originally related to Freud’s conception of artmaking as the expression of repressed content in neurotics who are dealing with powerful pressures originating in the Id [8], and evidence that artists cope with their distress through art, e.g., [9,10,11,12]. Findings from studies on the association between mental distress and the drive to engage in artmaking, e.g., [13,14,15] also support this assumption.

Other theorists and researchers [16,17,18] argue that playfulness, creativity and artistic endeavor are more closely related to well-being than to distress. Winnicott, for example, considered that artmaking, creativity and playfulness are the visible counterparts of mental health. These manifest in the encounter between the inner world and outer experience, and contain elements from both worlds, such as emotions, experiences, and inner fantasies, all of which are expressed and can be processed in the real world [19]. According to this view, well-being engenders greater playfulness and creativity, as also found in artmaking. Similarly, attachment theorists have suggested that exploratory and playful activity which are part of the artistic endeavor [19,20] are enabled when there is a sense of security that derives from secure attachment, and an ability for emotional regulation. For example, as can be seen in the Strange Situation [21], exploratory activity is delayed until emotional regulation of distress is achieved, which allows the child to return to play [22]. A number of studies examining the relationship between creativity and playfulness, as well as mental well-being and the ability to regulate emotions, lend weight to this assumption, e.g., [23,24]. For example, in a study of 61 kindergarten to fourth grade girls, Hoffmann and Russ found that participants who were better able to manage their emotions were also more comfortable engaging in the play task and showed higher levels of imagination and organization while playing [25]. A study that examined the expression of attachment patterns among adults who played the mirror game found that individuals with secure classification expressed more ease and flow in their movements [26].

These differences in interpretations of the nature of relationship between artmaking, creativity and mental state are probably related to the complexity of this relationship [27,28,29,30,31]. Researchers and theorists have stressed the importance of the definition of mental distress, its characterization [32,33] and intensity [34], in the process of understanding the association between these variables. The characteristics of the association between these two variables are also related to definitions of creativity and artistic endeavor, since these are close but not identical concepts, and different studies use different operationalizations to evaluate and measure them [29,31]. Finally, the way in which the relationship between the variables is investigated also plays a role in the variability in the association [35,36,37].

The different associations between the artmaker’s mental state and the experience of artmaking in various situations also raises questions about the role of artmaking in the therapeutic process with children. Drawing on the theoretical idea that artmaking stems from distress, children who are sent for treatment for emotional distress and behavioral problems are assumed to be attracted to artmaking. By contrast, the notion that artmaking is an expression of wellbeing suggests that clients need to feel relaxation, security and well-being before they can engage, enjoy and express themselves through art materials.

The present study examined the relationship between the behavioral manifestations of children referred to art therapy in the education system before the start of treatment, and their responses to artmaking over the course of treatment.

### 1.2. Artmaking during Therapy

This study also examined whether treatment outcome could be predicted through assessments of the child’s experiences of artmaking during therapy. The theoretical premise of a relationship between artmaking and distress implies that artmaking can provide support and can contribute to enhancing the artmaker’s mental well-being, e.g., [2,3,38]. In children, artmaking provides a fun and natural way to express content from their inner world [1,39,40,41,42,43]. The inviting presence of the art materials and the enjoyable opportunity to create establish a safe space for the development of the therapeutic relationship [42]. The art materials and the blank sheet of paper transform into an esthetic expression of the artmaker’s inner world, while containing the pain. Observation of the esthetics of the artistic creation conveys the message that the clients’ painful experience can be thought about and contained [1]. This externalization of painful experiences, and their organization by the artmaker in ways exterior to the self within the artistic space also impact the artmakers, who can then reflect on their work [44]. Consistent with these claims, there is growing evidence for the effectiveness of art therapy for children, e.g., [3,41,45,46].

Within this process, the positive and pleasurable aspects of artmaking appear to play an important role. Artmaking can produce a positive experience and engender positive emotions [47,48,49]. Art therapy clinicians, e.g., [47,50,51,52,53,54] have reported that the positive features involved in artmaking enables artmakers to experience ‘flow,’ an experience of full involvement, enjoyment and focus on the process [55,56]. Positive psychology posits that the experience of flow and positive emotions such as pleasure, interest, hope and love enable the continued organization of the self and psychological growth [57,58]. Art therapists suggest that esthetic, creative, and enjoyable experiences are often an essential component of creative developmental processes [59] because they provide the artmakers with the motivation to cope and bear the painful parts of therapy, and explore themselves in a positive and enjoyable way [60]. The notion that a positive experience of art in therapy would have a positive impact on treatment success was explored in this study by examining whether children’s experiences of art during therapy could serve as a predictor of improvement in school art therapy outcomes.

### 1.3. The Current Study

This study examined the relationship between the experience of artmaking in art therapy, and the child’s condition in the context of art therapy in the school setting. Art therapy in educational settings has developed exponentially in recent years [46,61]. In Israel, art therapists work in almost every educational setting, thus making treatment accessible to many children who would not otherwise have access to therapy [62]. School art therapy is intended to support children’s integration in school, and their ability to engage in the learning, educational and social activities that take place in the educational framework. School art therapy has the further advantage of teamwork made possible by treating clients in the location where they are studying [63]. To include the educational context in which the treatment took place, the children’s emotional conditions were assessed by their teachers and parents reports on behavioral problems before and after therapy. A comprehensive literature review on thirty-seven studies from 2020, showed that art therapy for children and adolescents with psychosocial problems can lead to improvement regardless of the type of art materials and therapeutic intervention method [64]. The children’s experience of artmaking in art therapy was measured using a self-report questionnaire that includes a number of questions about the respondent’s experience in the last therapy session [65].

Two hypotheses were formulated: 1. Parents’ and teachers’ reports of behavioral problems before treatment would be inversely correlated with experiences of artmaking in art therapy after the first month of therapy. 2. Improvement in the child’s condition, as assessed by differences in parents’ and teachers’ reports on behavioral problems before and after therapy could be predicted from the child’s experience of artmaking in art therapy, i.e., more positive responses to art would predict higher gain scores.

## 2. Materials and Methods

This quantitative longitudinal study is part of a broader project examining art therapies in the education system from 2015 to 2017 to better characterize the processes involved [66,67,68,69,70], the conditions for treatment success [71] and therapists’ well-being [72]. Since this study aimed to capture a naturalistic setting, it should be seen as pseudo-experimental.

### 2.1. Participants

Seventy-seven art therapy clients in the Israeli elementary school system, their home-room teachers and their parents completed self-report questionnaires during one year of therapy. The students involved were all eligible for special education services in regular education (integration hours) or as part of a special education class in regular education, and were referred for treatment in the school setting. None were receiving any other form of additional psychotherapy. They were treated by 48 art therapists who consented to participate in the study for one year. The sample was composed of 55 boys and 22 girls enrolled in first to seventh grade. They ranged in age from seven to 13 (*M* = 10.07, *SD* = 1.53). Forty-six were defined as middle SES, 19 as low SES, and nine high SES. Sixty-eight were Jewish and nine were Muslim; 28 received one-on-one therapy, 36 were in group therapy, nine in a dyadic setting, and for four, no type of setting was noted. The referrals for therapy were typical to the school system in general, and consisted of social difficulties, emotional difficulties, low self-esteem, behavioral disorders and attention deficit disorders that impaired the students’ ability to learn.

### 2.2. Measures

#### 2.2.1. Art-Based Intervention Questionnaire 

This self-report questionnaire [65] examines individuals’ creative experiences working with art materials. The respondents indicate the extent to which each Likert-type statement corresponds to their experience with art materials on a scale of 1–7. The 41 original items cover four categories, with 10 subscales: 1. Feeling and thoughts preceding the artistic process, which includes the positive excitement, confidence, and aversion subscales; 2. Feeling and thoughts during the artistic process, which includes the pleasantness and therapeutic value, competence, difficulty in carrying out the artistic task, and playfulness subscales; 3. Attitudes toward the artistic product, which has one subscale; and 4. Attitude toward the material, which includes the meaningful and pleasantness subscales. In the current study, a short 15-item version adapted to children with items from categories 2 and 3 was used. The ABI was administered to the children at the start (T1), middle (T2) and the end of one school year of therapy (T3). Table 1 lists the 15 items.

A high score on this scale expresses a positive experience and enjoyment of artmaking. Good reliability of the subscales has been reported by the authors ranging from 0.45 to 0.91, with a reliability of 0.91 for the overall score [64]. In the present study, the reliability of the overall score was found to be good on all three measurements (αT1 = 0.73; αT2 = 0.75; αT3 = 0.75).

#### 2.2.2. Child Behavior Checklist 

The Hebrew version of the two scales was administered. It evaluates students’ behavior and functioning, as well as changes over time and/or after the intervention. The Teacher’s Report Form (TRF) is made up of 113 items. The parents’ form, the Child Behavior Checklist (CBCL), is made up of 109 items. Respondents circle “not true of him/her” (0), “sometimes true” (1), or “always true of him/her” (2) for each item to indicate the presence/absence of the problem. The items are divided into eight subscales in both checklists. These can be grouped into three larger subscales: internalizing problems (the anxiety/depression, introversion/depression, and somatic complaints subscales), mixed problems (the social problems, cognitive problems, and attention problems subscales) and externalizing problems (the aggressive behavior and delinquent behavior subscales) [73]. In the current study, only the composite scales (internalizing problems, mixed problems, and externalizing problems) were administered. The Child Behavior Checklist was filled in by the homeroom teachers and parents at the beginning of the year (pre-intervention questionnaires), and at the end of the year at the conclusion of therapy (post-intervention questionnaires). The Cronbach’s alphas for the homeroom teachers’ and parents’ scales, pre- and post-intervention, ranged from 0.61 to 0.95.

The gain scores were calculated as the pre-score minus the post score to obtain a positive different index: the higher the difference score, the more positive change between the first and second measurement.

### 2.3. Procedure

Therapists were contacted to take part in the study and the Israeli Association for Creative Arts Therapies (YAHAT) posted calls to participate. Art therapists were asked to select one or two of their 1st to 7th grade clients and their parents who had consented to take part. The data were collected on Qualtrics software. A research assistant helped the students fill in the questionnaires after coordination with the school and the therapist. The teachers and parents filled out the questionnaires after receiving a link from the experimenter at the beginning of the year, and again at the end of the treatment.

### 2.4. Professional Ethics and Confidentiality

The therapists and parents were informed by the consent form that they could withdraw from the study at any time and that withdrawal would have no detrimental effects. The students were informed orally of their rights and that withdrawal would have no effect on the continuation of therapy. In order to maintain anonymity, each client was given a code that was used on the questionnaires for identification purposes.

## 3. Results

### 3.1. Preliminary Analysis

Examination of the data distribution showed that most of the ABI, CBCL and TRF scales were normally distributed. Calculation of the difference between pre-therapy measurement and post-therapy measurement on the TRF and CBCL showed that the gain scores of the outcome measures were positive, but low. Thus, it may be said that there had been some, but not major improvement in the outcome measures following treatment.

### 3.2. Correlations between Children’s Behavioral Problems Prior to Therapy and Their Evaluation of Artmaking in Therapy

The correlations between the children’s behavioral problems before starting treatment as reported by the parents’ and teachers’ assessments, and the children’s responses to artmaking were calculated using Pearson’s *r*, with a Bonferroni correction for multiple correlations.

As shown in Table 2, there was a significant inverse correlation between clients’ response to artmaking at T1 and the parents’ and the teachers’ assessment of externalizing problems (*r*, CBCL = −0.374, *p* < 0.002; *r*, TRF = −0.409, *p* < 0.001), and mixed problems (*r*, CBCL = −0.313, *p* < 0.012; *r*, TRF = −0.361, *p* < 0.003). In other words, the more the children were characterized as having externalizing and mixed problems by their parents and teachers, the less positively they perceived the experience of artmaking at T1, after one month of therapy. By contrast there were no significant correlations between parents’ and teachers’ reports of the children’s condition prior to therapy on these scales and the children’s responses to artmaking in the middle and towards the end of therapy (T2, T3), or for the parents’ and teachers’ reports of internalized problems before treatment and the children’s responses to artmaking at any of three time points.

### 3.3. Predicting Improvement in Outcome Measures through Responses to Artmaking

To test the second hypothesis, a linear regression was calculated to predict the gain scores using the ABI score at each time point. This test was run six times, to test the ability to predict the internalizing problems, externalizing problems and mixed problems separately for parents’ and teachers’ reports. To correct for the effect of each child’s initial condition, the pre- measurement score in the predicted index was also entered into the predictors.

The regression model for predicting gain scores in teachers’ reports of internalizing problems was significant [F (4,56) = 6.21, *p* = 0.000] and explained 30.7% of the variance in improvement in internalizing problems. Aside from internalizing problems before starting treatment, whose contribution to the equation was predictable but non-significant with respect to the research questions, the only other significant regression predictor was the child’s response to art at T1 (β = 0.335, *p* = 0.014).

In the regressions to predict gain scores from the parents’ reports of internalizing, mixed, and externalizing problems, and teachers’ report of mixed and externalizing problems, the only factor that contributed to the significance of the model was the pre-measurement score in the predicted index.

## 4. Discussion

This study examined the relationship between children’s experiences of artmaking in art therapy, and behavioral problems as reported by the clients’ parents and teachers. The hypotheses as to the possible correlations between children’s behavioral problems as reported by parents and teachers before treatment and the child experience of artmaking in therapy, and with respect to the ability to predict improvement in behavioral problems based on the children’s experience of doing art in therapy, were partially confirmed.

In terms of H1, there was a negative correlation between children’s externalizing problems and mixed problems before treatment, as reported by both teachers and parents, and children’s experience of artmaking at T1, after approximately one month of therapy. Specifically, the more social problems, cognitive problems, attention problems, aggressive behavior and delinquent behavior were described by parents and teachers, the less enjoyment the children reported in artmaking at T1, about a month after the start of treatment. This correlation between measurements from two different sources that were blind to each other’s responses may hint at a possible association between clients’ mental states and their experiences with art materials, and support the theoretical assumption that mental well-being and emotional regulation [19,22] impact the ability to enjoy creative and artistic pursuits. This idea is consistent with findings suggesting that creativity is enhanced by positive mood states [23,74].

However, the correlation between children’s behavioral problems before treatment and responses to artmaking during the first month of treatment was unrelated to internalizing problems, based on the parents’ and teachers’ reports. In other words, the presence or absence of anxiety/depression, introversion/depression, and somatic complaints were not related to the children’s experience of working with art materials in therapy. This may support previous claims as to the importance to precisely define mental state when examining its association with and responses to artmaking [32,33].

The findings can be interpreted as indicating that children with externalizing problems may feel that working with art materials is challenging because it can require siting on a chair and focusing; therefore, the greater their distress, the less they enjoy engaging in artmaking. On the other hand, it is possible that treatment that involves more physical activities and the release of body tension and movement would be more appealing for these children [75]. This is in line with an Iranian study of 30 adolescent girls aged 14–18 who were given 6 sessions of art therapy. These researchers reported a statistically significant improvement in the 15 girls with internalizing problems compared to the control group, but no improvement in the 15 girls with externalizing problems [76]. However, alongside this possible explanation, it is worth noting that there is also ample evidence of the power of art when working with children with externalizing problems, e.g., [43,77,78]. Furthermore, since art therapists in the education system often work under inappropriate conditions which do not always allow for movement, or a regressive and liberating use of materials [62,71], they may not be able to intervene in an appropriate manner to deal with children with externalized behavior problems.

The differences in correlations between children with externalizing and internalizing behavioral problems and their response to artmaking in therapy may also be related to their overall scholastic experiences. Studies show that in primary school, externalizing behaviors have been systematically found to be associated with lower levels of behavioral engagement in school activities [79,80]. This raises the possibility that art making in therapy in children with externalizing problems may be experienced as another unwanted activity in school, which may explain the decline in enjoyment of art in therapy as the level of externalizing problems of this type increases. In contrast, for children dealing with internalizing experiences of depression and/or anxiety, emotional distress may not influence the experience of artmaking, and other factors such as the relationship with the therapist, love of art, articulation, or artistic talent could have a greater impact on their artistic experience in therapy.

No correlation was found between behavioral problems in the parents’ and teachers’ reports prior to therapy and the experience of artmaking in the middle and towards the end of therapy, at time points 2 and 3. It is likely that as therapy progressed, other factors influenced the way the children experienced the artistic endeavor. For example, the learning of the artistic language over the course of treatment may have had an effect on the degree of enjoyment of the artistic activity. Alternatively, children’s state may have gradually improved as a result of therapy, and their reaction to the artwork may have been influenced by their current condition and not the condition at the start of the treatment. Another possibility is that the developing therapeutic alliance with the therapist acted as an additional factor that affected motivation for artmaking, and the enjoyment of the experience. A previous study reported a positive relationship between therapeutic alliance and response to artwork in a simulation in which 34 female students played the role of therapist, and 37 female students played the role of the clients. The findings showed that the stronger the therapeutic alliance, the more positive the client’s response to the creative experience and attitude towards the artistic product [81]. The relationship between therapeutic alliance and response to artmaking in art therapy has not yet been investigated in children, but for children the strengthening of the therapeutic alliance with the therapist throughout the year may become a motivating factor influencing the creative experience.

In terms of H2, the regression model predicting the gain scores in the internalizing problems indices, as assessed from the teachers’ reports and the children’s responses to artmaking at the three time points was significant, and explained a third of the variance in child improvement. The regression predictor that was found to be significant was the child’s experience of artmaking at Time 1. This finding is interesting in light of the fact that the children’s response to artmaking was not correlated with the children’s condition in terms of internalizing problems at the beginning of treatment. It is possible that for these children, artmaking as a form of self-expression of the inner psychic world and content has a relatively greater value that can have a positive effect on their mental state and thus on the success of treatment. This finding supports the assumption of the theoretical concept of flow, regarding the beneficial value of enjoyment from creating, and focuses on the value of the positive experience of artmaking for children with internalizing problems [57,58].

The fact that the art experience at T2 and T3 was unrelated to changes in the clients’ internalizing problems may suggest that the initial experience of artmaking at the beginning of therapy, which is less affected by the development of the therapeutic alliance and treatment, may provide more salient information about the relationship between the experience of art making in therapy and improvement in mental well-being. These issues call for further research.

## 5. Conclusions

These findings show that the critical importance of gaining a better understanding of the potential associations between externalizing and mixed behavioral problems and responses to artmaking. Along with maintaining that art can be a beneficial tool in therapy for anyone and everywhere [82] art therapists also concede that occasionally, the use of art materials in therapy can be challenging, as some children have difficulties engaging in the artistic process. This raises questions of how to encourage children to be involved in art, which sometimes ends in a referral to a therapist working in another medium (e.g., drama), particularly in schools where there are therapists with different specialties. Understanding that a negative experience working with art materials may be related to the severity and nature of the child’s behavioral problems may help cope with this challenge. In addition, evidence from previous studies suggests that this relationship has two facets, and that enjoyment of the art in therapy may become a positive anchor that has a positive effect on the child’s experience at school [83].

The findings with respect to the relationship between the experience of engaging in artmaking in therapy and an improvement in internalizing problems, depression, anxiety and somatic problems following treatment underscore the importance of a positive experience of artmaking and raise interesting questions about the factors that create this positive experience. Future work should concentrate on these questions.

## 6. Limitations and Suggestions for Further Research

This preliminary longitudinal study points to possible associations between the experience of artmaking in therapy and the amelioration of elementary school children’s behavioral problems. However, to understand the nature of this relationship, further investigations are needed. Studies could examine a larger sample, address other intervening variables that can account for the relationship between the experience of artmaking in school art therapy, and behavioral problems and the improvement of behavioral problems after treatment. These could be related to mental well-being, the school experience, the child’s involvement in school activities, the development of the therapeutic alliance, attitude towards art in general, and others. In addition, this study did not administer sub-scales of the CBCL or the TRF, which might have provided a sharper perspective on the relationship between the variables. The experience of artmaking was investigated as a single variable, without taking the components that constitute the artistic experience and its development into account, or the therapists’ point of view, which future works could assess through observational and interview-based research. Since psychotherapists and art therapists’ conceptualization, theoretical knowledge and understanding are critical to understanding change processes in therapy [84,85], future research should include interviews with art therapists in schools, who can provide new insights on how mechanisms of change differ among children coping with externalizing problems, children coping with internalizing problems and children coping with mixed problems.

## Figures and Tables

**Table 1:** ABI-child version.

ABI Items
I felt I could keep on going for hours
I felt I was being creative
I had a difficult time executing my ideas *
I felt I was good at this kind of activity
I felt I needed to make a considerable effort *
I encountered lots of technical difficulties in performing the artistic task *
I had a hard time sitting still and wanted to get up and move around *
I enjoyed working on my art project
I found it pleasant to be creating something
Working on my art project, I felt a sense of inner peace and warmth
I felt it was OK to make mistakes
I wanted to keep what I had made
I was excited to see what I had created
I was surprised by what I had made
I wasn’t satisfied with what I had made

* reverse scored.

**Table 2:** Matrix correlates between response to artmaking and behavioral problems.

Children’s Condition before Therapy		ABI—Children’s Artmaking Experience		
		T1	T2	T3
TRF	Externalizing problems	−0.409 * p < 0.001	−0.311 *p* < 0.012	−0.194 *p* < 0.127
	Internalizing problems	−0.153 *p* < 0.223	−0.146 *p* < 0.246	−0.069 *p* < 0.591
	Mixed problems	−0.361 * p < 0.003	−0.240 *p* < 0.054	−0.175 *p* < 0.171
CBCL	Externalizing problems	−0.374 * p < 0.002	−0.192 *p* < 0.128	−0.249 *p* < 0.049
	Internalizing problems	−0.251 *p* < 0.046	−0.103 *p* < 0.416	−0.034 *p* < 0.792
	Mixed problems	−0.313 * p < 0.012	−0.141 *p* < 0.266	0.009 *p* < 0.943

* *p* < 0.016.

## Data Availability

The datasets for this manuscript are not publicly available to protect participants’ confidentiality.
